# EEG alpha peak frequency: cognitive impairment severity marker in isolated REM sleep behavior disorder

**DOI:** 10.1038/s41531-025-01059-z

**Published:** 2025-07-01

**Authors:** Sophia Schopp, Jan de Zeeuw, Sophia Stotz, Martin Haberecht, Katy Sahra Weihrich, Michail Plotkin, Frederik Bes, Dieter Kunz

**Affiliations:** 1 Clinic for Sleep & Chronomedicine, St. Hedwig-Hospital, Berlin, Germany; 2 Freie Universität Berlin, Berlin, Germany; 3 Sleep Research & Clinical Chronobiology, Institute of Physiology, Charité–Universitätsmedizin Berlin, Berlin, Germany; 4 Institute of Nuclear Medicine, Vivantes Hospitals, Berlin, Germany

**Keywords:** Parkinson's disease, Parkinson's disease

## Abstract

Isolated REM sleep behavior disorder (iRBD) is a prodrome of α-synucleinopathies like Parkinson’s disease (PD) and dementia with Lewy bodies (DLB). The best marker for prediction of lead time to phenoconversion is reduced striatal dopamine transporter (DaT)-binding, unavailable for most patients. This study investigated EEG alpha peak frequency (APF) slowing – an established marker of cognitive deterioration – as a severity marker in iRBD patients. In 320 patients clinically suspected of RBD 3-night-polysomnography was performed. After exclusion of 131 patients, mainly due to psychotropic medication, three groups were studied: Non-Syn – motor behavior unrelated to RBD (*n* = 34); iRBD (*n* = 122); RBD converted to overt α-synucleinopathies (PD = 19; DLB = 14). Data show in patients with iRBD significant correlations between APF, DaT-binding ratios and cognition. The strong correlation of APF < 8 Hz with caudate DaT-binding (*r* = 0.50–0.65) suggests, APF could be an easy-to-use severity marker for counseling patients on lead time until possible conversion to overt *α*-synucleinopathy especially in DLB.

## Introduction

Isolated rapid eye movement (REM) sleep behavior disorder (iRBD) is characterized by the loss of muscle atonia during REM sleep (REM sleep without atonia; RWA), causing patients to act out their dreams through vocalization and complex behaviors such as yelling or fighting^1^. This sleep disturbance occurs in about one percent of the general population above the age of 60 years^2^. Information online often alerts these individuals to the association between iRBD and neurodegeneration, with studies showing that over 95% of patients with iRBD convert to overt synucleinopathy within 14 years of an iRBD diagnosis^3,4^.

iRBD has been recognized as a prodromal state of α-synucleinopathies such as Parkinson’s disease (PD), dementia with Lewy bodies (DLB) and multiple system atrophy^4,5^. Naturally, many patients are concerned about the lead time until clinical signs of parkinsonism and dementia occur, as well as the implications for their future personal and medical management. At present, one of the most reliable prognostic markers for synucleinopathic neurodegeneration is the availability of dopamine transporters (DaT) in the basal ganglia, which can be measured using single-photon emission computed tomography (SPECT)^6^. Impairments in various DaT-SPECT regions have been linked to different clinical manifestations of α-synucleinopathies, with lower DaT binding observed in the caudate nucleus of patients with DLB compared to those with PD^7^. While the putamen is primarily involved in motor function in PD, the caudate nucleus plays an important role in cognition^8^. DaT-SPECT imaging, however, is expensive, not widely available, and requires the injection of radioactive substances. Thus, there is an unmet need for accessible and easy-to-use biomarkers that can estimate the lead time from iRBD to the phenoconversion to clinical parkinsonism or dementia.

A slowing of the dominant frequency in the electroencephalogram (EEG) has long been known to be associated with cognitive impairment and neurodegeneration, particularly in DLB or PD with dementia^9,10^. Given that polysomnographic sleep recordings (PSGs) are essential for the diagnosis of iRBD, the dominant EEG frequency can be assessed without additional procedures. Several studies have shown that EEG slowing also correlates with cognitive impairment in patients with iRBD^11–14^. However, these studies were performed using EEG power ratios. A much simpler approach would be to assess the dominant peak frequency in the EEG theta/alpha range.

The aim of this study was to investigate the associations between alpha peak frequency (APF) during waking EEG in PSG and (a) DaT-binding ratios as well as (b) neurocognitive function in a large single-center cohort of patients with iRBD.

## Results

### Patients

**Fig. 1:**
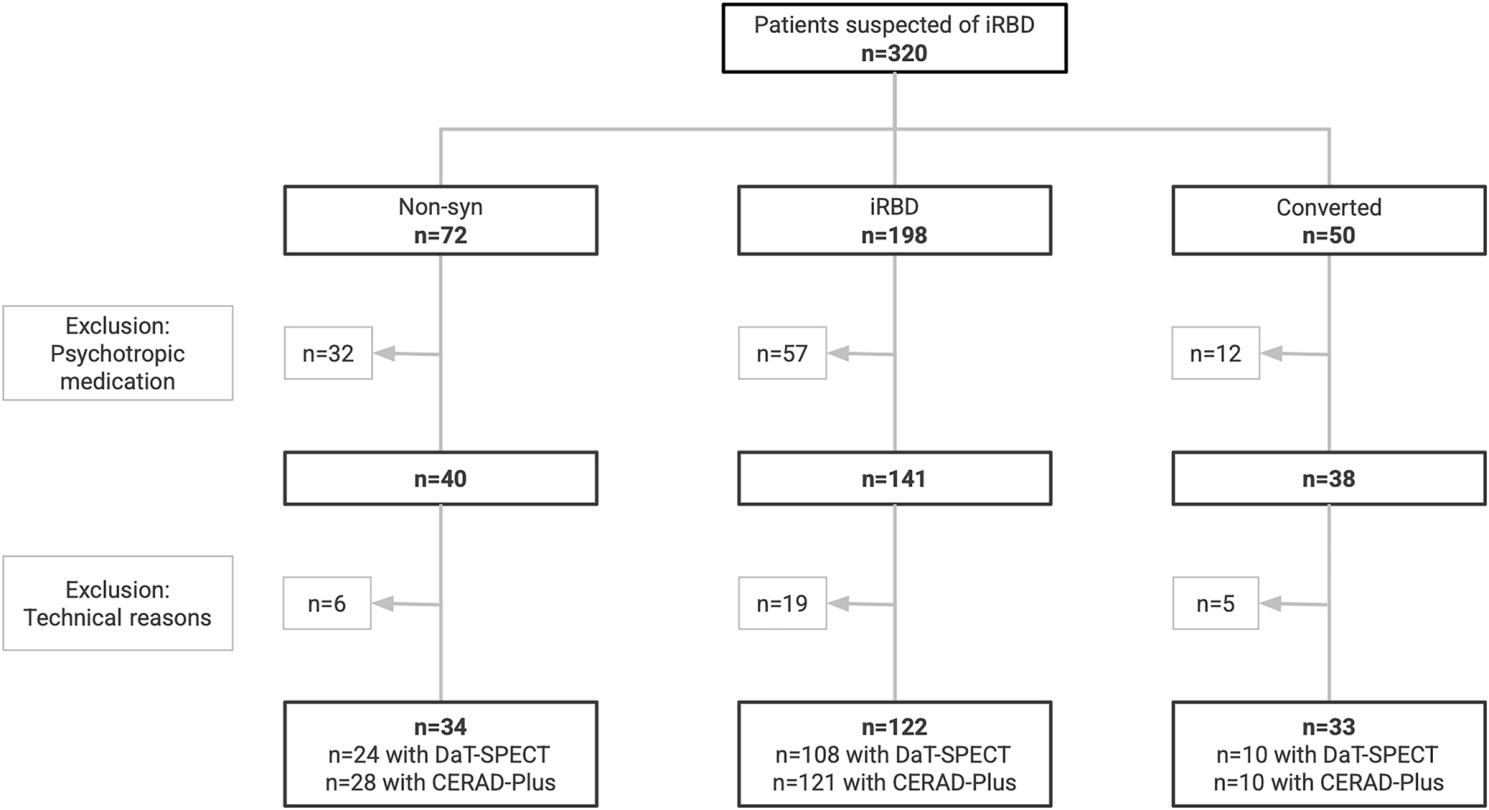
Flowchart of patient inclusion and exclusion.

Out of 320 consecutive patients clinically suspected of having RBD, 198 were diagnosed with iRBD, 14 with secondary RBD, 50 with RBD associated with overt α-synucleinopathy, and 58 with motor behavior during sleep not attributable to RBD (non-synucleinopathy control group; Non-Syn; see flowchart of Fig. 1).

Of the 198 patients with iRBD (seen between 2016 and 2024), 57 were excluded due to the use of psychotropic substances (antidepressants: *n* = 37; dopaminergic substances: *n* = 7^15^; other drugs: *n* = 13), and 19 were excluded due to technical reasons (absence of an alpha rhythm: *n* = 3; compliance issues: *n* = 2; tumor: *n* = 1; and technical difficulties: *n* = 13). Among the 122 patients with iRBD who were included, 108 underwent DaT-SPECT imaging and 121 completed a cognitive assessment with the CERAD-Plus test battery.

The same exclusion criteria were used for the Non-Syn group, resulting in the exclusion of patients due to psychotropic substance use (*n* = 18) and technical reasons (*n* = 6). Of the 34 remaining Non-Syn patients, 24 underwent DaT-SPECT imaging and 28 completed CERAD-Plus cognitive assessment. Correlations between APF and DaT-SPECT or CERAD-Plus results were analyzed for the total cohort, as well as for subgroups of patients in a more advanced stage of the disease, identified based on DaT-SPECT cut-offs.

To evaluate patients who had converted to overt α-synucleinopathies, we expanded this group to all such patients diagnosed with RBD in our clinic since 2004. Out of a total of 50 converted patients (26 with PD and 24 with DLB), 12 were excluded due to the use of psychotropic medication and 5 due to technical reasons. Thus, 33 patients who had converted to overt *α*-synucleinopathies (19 with PD and 14 with DLB) were included in the APF analysis. Of these 33 RBD-converted patients 10 had a DaT-SPECT and 10 CERAD-Plus.

Because the literature suggests that cognition is primarily associated with the caudate region^16–20^, we hypothesized that the APF would show the strongest correlation with DaT binding in this region. Therefore, subgroup analyses were performed in patients who had at least one DaT-binding *z*-value in the caudate. These patients were stratified as follows: those with a *z*-value greater than −1.0 (*n* = 46), between −1.0 and −2.0 (*n* = 44), and less than −2.0 (*n* = 19), indicating increasing levels of pathological severity.

### Alpha peak frequency

Table 1 shows the demographics and the APF of the Non-Syn patients, patients with iRBD as the total cohort, those without a DaT-SPECT and divided in three subgroups based on the degree of DaT reduction in the most affected caudate, as well as the converted patients with PD and DLB. The Non-Syn patients had the highest APF (9.09 Hz). The patients with iRBD had slightly lower APFs with the lowest APF in the subgroup with the more advanced DaT-SPECT abnormalities (i.e., caudate *z*-value ≤−2; APF 8.32 Hz). Patients who had converted to overt *α*-synucleinopathies (*n* = 33) had a significantly lower APF (PD: 7.57 ± 1.07 Hz; DLB: 6.88 ± 0.78 Hz) compared to Non-Syn and iRBD patients (*p* < 0.001).

**Table 1:** Demographics, EEG alpha peak frequency (APF) & DaT-binding in α-synucleinopathy

		Non-Syn	iRBD					Overt alpha-synucleinopathy	
			Total	No DaT-SPECT)	DaT-SPECT *n* most affected caudate z-value				
					>−1.0	≤−1.0 to >−2.0	≤−2.0	PD	DLB
		(*n* = 34)	(*n* *=* 122)	(*n* = 14)	(*n* = 46)	(*n* = 44)	(*n* = 18)	(*n* = 19)	(*n* = 14)
**APF (Hz)**	**Mean (±SD)**	**9.09 (±0.96)**	**8.78 (±0.91)**	**8.95 (±1.01)**	**8.81 (±0.86)**	**8.87 (±0.91)**	**8.32 (±0.89)**	**7.57 (±1.07)**	**6.88 (±0.78)**
Age (years)	Mean (±SD)	63.4 (±13.0)	69.9 (±7.6)	69.0 (±10.7)	69.4 (±7.3)	69.5 (±6.8)	72.2 (±7.1)	71.8 (±8.5)	73.9 (±5.8)
Male	%	79	80	71	76	82	89	79	100
DaT-SPECT path.^a^	%	0	21	-	2	23	61	88	50
MDS-UPDRS-III	Mean(±SD)	1.7 ( ± 1.9)	1.3 ( ± 1.9)	2.5 ( ± 2.6)	1.2 ( ± 2.0)	1.0 ( ± 1.6)	1.2 ( ± 1.3)	10.8 ( ± 9.0)	10.5 ( ± 9.9)
Anosmia	%	4	38	42	33	29	73	64	75
Constipation	%	28	39	50	22	47	56	70	60
TMT-A z-value **≤**−2	%	3	3	0	5	0	6	31	33
TMT-B z-value **≤**−2	%	0	0	0	0	0	0	25	50

DaT-SPECT caudate, primarily involved in cognition, was used to create subgroups of patients with iRBD; *iRBD* isolated REM sleep behavior disorder, *PD* Parkinson's disease, *DLB* dementia with Lewy bodies, *DaT-SPECT* dopamine transporter single-photon emission computed tomography, *Non-Syn* Non-Synucleinopathy, *MDS-UPDRS-III* Movement Disorders Society - Unified Parkinson Disease Rating Scale, *TMT* Trail Making Test; TMT-A: specific for attention; TMT-B: specific for executive functioning; olfaction = Sniffin’ Sticks; constipation = Scales for Outcomes in Parkinson’s disease (SCOPA); ^a^pathologic according to expert interpretation.

A negative correlation was observed in patients with RBD between age and APF (*r* = −0.353; *p* < 0.001). The patients with DLB and the iRBD subgroup with the more advanced DaT-SPECT were significantly older than the Non-Syn patients (*p* < 0.029), while the other (sub)groups did not differ significantly in age.

### Mean DaT-SPECT values

The mean (±SD) DaT-SPECT values of Non-Syn patients and patients with RBD are presented in Table 2. Patients with RBD showed 12–21% lower DaT-binding SBRs, significantly reduced in all DaT-SPECT regions (*p* < 0.003), compared to Non-Syn patients.

**Table 2:** DaT-SPECT & CERAD values

	Non-Syn controls		RBD	Non-Syn controls		RBD
DaT-SPECT region	(*n* = 24)		(*n* = 118)	(*n* = 24)		(*n* = 118)
	SBR			*z*-value		
	mean (±SD)	*p*-values	mean (±SD)	mean (±SD)	*p*-values	mean (±SD)
Right caudate	3.12 ( ± 0.30)	**<0.001**	2.73 ( ± 0.51)	−0.36 ( ± 0.77)	**0.007**	−1.06 ( ± 1.19)
Left caudate	3.24 ( ± 0.36)	**<0.001**	2.87 ( ± 0.48)	−0.22 ( ± 0.90)	**0.007**	−0.89 ( ± 1.12)
Right anterior putamen	3.16 ( ± 0.43)	**<0.001**	2.67 ( ± 0.53)	−0.02 ( ± 0.99)	**0.001**	−0.91 ( ± 1.21)
Left anterior putamen	3.04 ( ± 0.36)	**0.003**	2.68 ( ± 0.56)	−0.27 ( ± 0.94)	**0.033**	−0.90 ( ± 1.37)
Right posterior putamen	2.57 ( ± 0.44)	**<0.001**	2.11 ( ± 0.60)	−0.78 ( ± 0.98)	**0.009**	−1.65 ( ± 1.46)
Left posterior putamen	2.66 ( ± 0.45)	**<0.001**	2.13 ( ± 0.57)	−0.28 ( ± 0.94)	**<0.001**	−1.24 ( ± 1.27)

CERAD tests	(*n* = 28)		(*n* = 131)	(*n* = 28)		(*n* = 131)
	Absolute value			*z*-value		
	mean (±SD)	*p*-values	mean (±SD)	mean (±SD)	*p*-values	mean (±SD)
Verbal Fluency	20.11 ( ± 5.29)	**0.011**	23.50 ( ± 6.55)	−0.75 ( ± 1.14)	**0.001**	0.16 ( ± 1.18)
BNT	14.43 ( ± 1.20)	0.540	14.58 ( ± 0.82)	0.07 ( ± 1.05)	0.065	0.40 ( ± 0.85)
MMSE	29.00 ( ± 1.25)	0.767	28.86 ( ± 1.62)	−0.37 ( ± 1.24)	0.261	−0.08 ( ± 1.15)
Wordlist Learning	20.96 ( ± 3.84)	0.388	20.21 ( ± 4.28)	−0.24 ( ± 1.07)	0.284	−0.07 ( ± 1.25)
Wordlist Recall	7.36 ( ± 2.90)	0.208	6.68 ( ± 2.09)	−0.31 ( ± 1.31)	0.628	−0.18 ( ± 1.14)
Wordlist Recognition	19.39 ( ± 0.96)	0.820	19.11 ( ± 2.60)	−0.20 ( ± 0.98)	0.137	0.03 ( ± 0.97)
Constructional Praxis	10.25 ( ± 1.32)	**0.030**	10.69 ( ± 0.90)	−0.27 ( ± 1.26)	**0.004**	0.34 ( ± 0.86)
Constructional Praxis Recall	9.68 ( ± 2.20)	0.990	9.74 ( ± 2.15)	−0.02 ( ± 1.30)	0.074	0.36 ( ± 1.22)
TMT-A	39.89 ( ± 17.09)	0.277	45.45 ( ± 22.22)	0.38 ( ± 1.31)	0.559	0.22 ( ± 1.26)
TMT-B	85.12 ( ± 30.49)	0.206	104.69 ( ± 54.88)	0.33 ( ± 1.32)	0.836	0.27 ( ± 1.29)
Phonetic Fluency	12.70 ( ± 4.67)	**0.045**	14.87 ( ± 5.15)	−0.26 ( ± 1.14)	**0.004**	0.42 ( ± 1.08)

Non-Syn = Non-Synucleinopathy patients, *iRBD* isolated REM sleep behavior disorder, *SBR* specific binding ratios, *BNT* Boston Naming Test, *MMSE* Mini-Mental State Examination, *TMT* Trail Making Test. Significant differences between Non-Syn and iRBD patients are indicated in bold (*p* < 0.05).

### APF and DaT-SPECT correlations

**Fig. 2:**
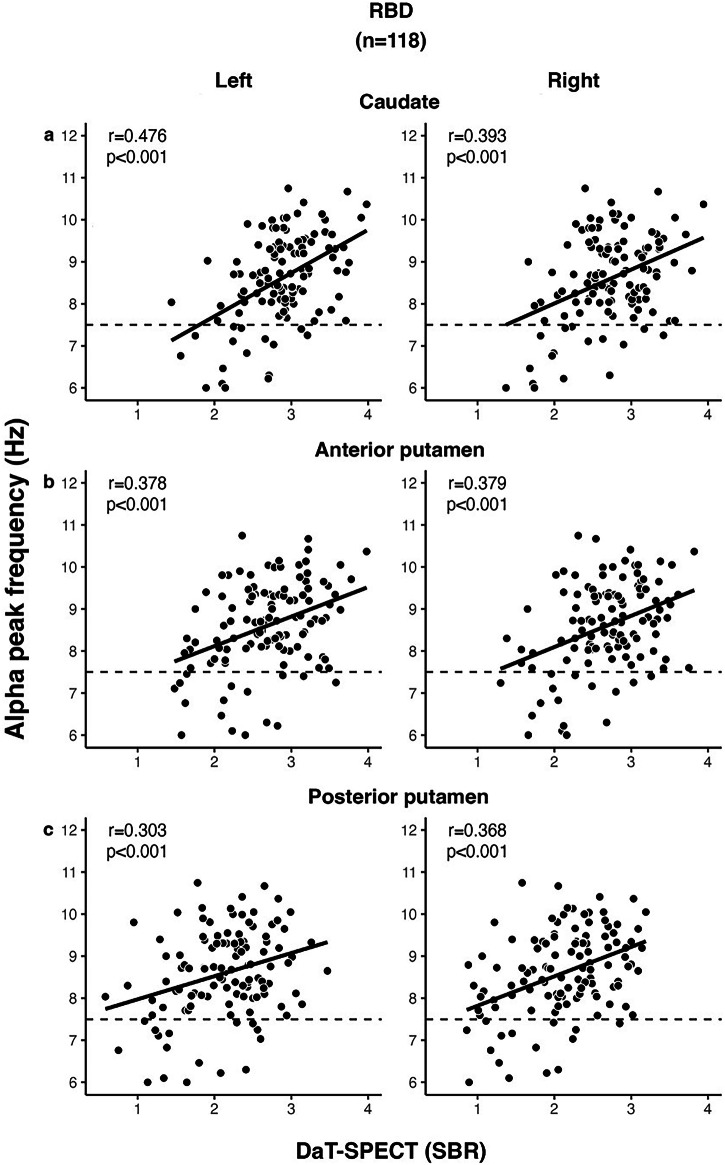
Correlation between APF and DaT-binding in RBD patients. In 118 included RBD patients DaT-SPECT was performed. APF correlated significantly with all DaT-SPECT regions (SBR-values): left and right **a** caudate; **b** anterior putamen; **c** posterior putamen.

Significant correlations were observed between APF and all DaT-SPECT regions (SBR values) in the total sample of included patients with RBD (i.e., patients with iRBD and RBD-converted patients) after Bonferroni-Holm correction for two tests (left and right regions; *p* < 0.050) (Fig. 2). The strongest correlation was observed between APF and the left caudate (SBR values: *r* = 0.476; *p* < 0.001; Fig. 2a).

**Fig. 3:**
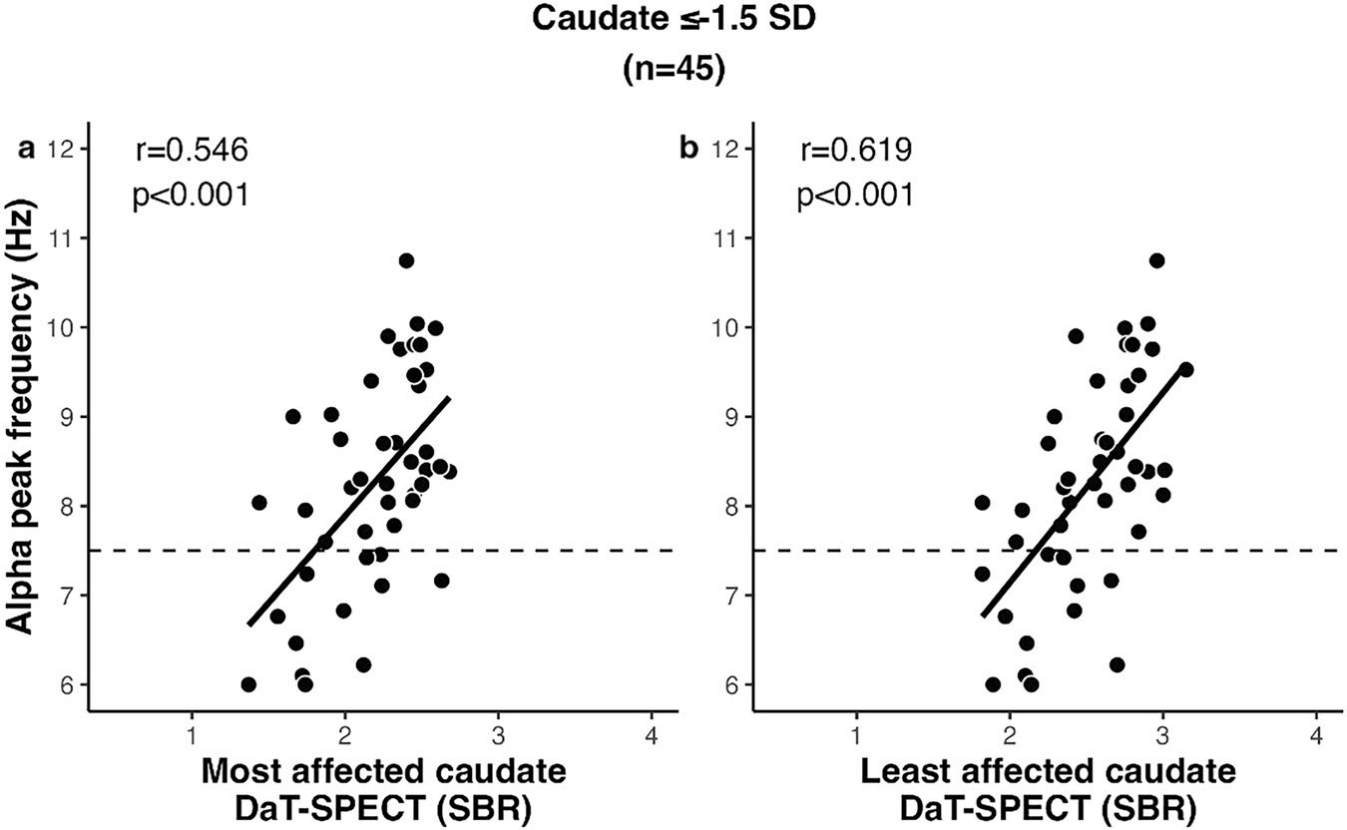
APF vs DaT-binding in advanced RBD patients. APF showed strongest correlation in advanced RBD patients who had a *z*-value of ≤−1.5 SD in one or both caudate regions (*n* = 45) in the **a** most affected caudate and **b** least affected caudate.

For RBD patients who had DaT-SPECT *z*-values of ≤−1.5 in the most affected caudate, a significant correlation between APF and DaT-SPECT was observed (SBR values: *r* = 0.546; *p* < 0.001; Fig. 3a), which was stronger than that observed in the total group of patients with RBD. The least affected caudate showed an even stronger correlation between APF and DaT-SPECT (SBR values: *r* = 0.619; *p* < 0.001; Fig. 3b).

Partial correlation analysis showed only minor changes, suggesting that the effect of age on the correlation between APF and DaT-SPECT is negligible. Among the 24 Non-Syn patients with DaT-SPECT, no significant correlations were found between APF and DaT-SPECT (*p* > 0.193).

### Mean CERAD-Plus values

The mean (±SD) CERAD-Plus test scores of Non-Syn patients and patients with RBD are shown in Table 2. Interestingly, cognitive test performance was, for the most part, comparable between groups, with some tests even showing better performance in patients with RBD compared to Non-Syn patients.

### APF and cognition correlation

**Fig. 4:**
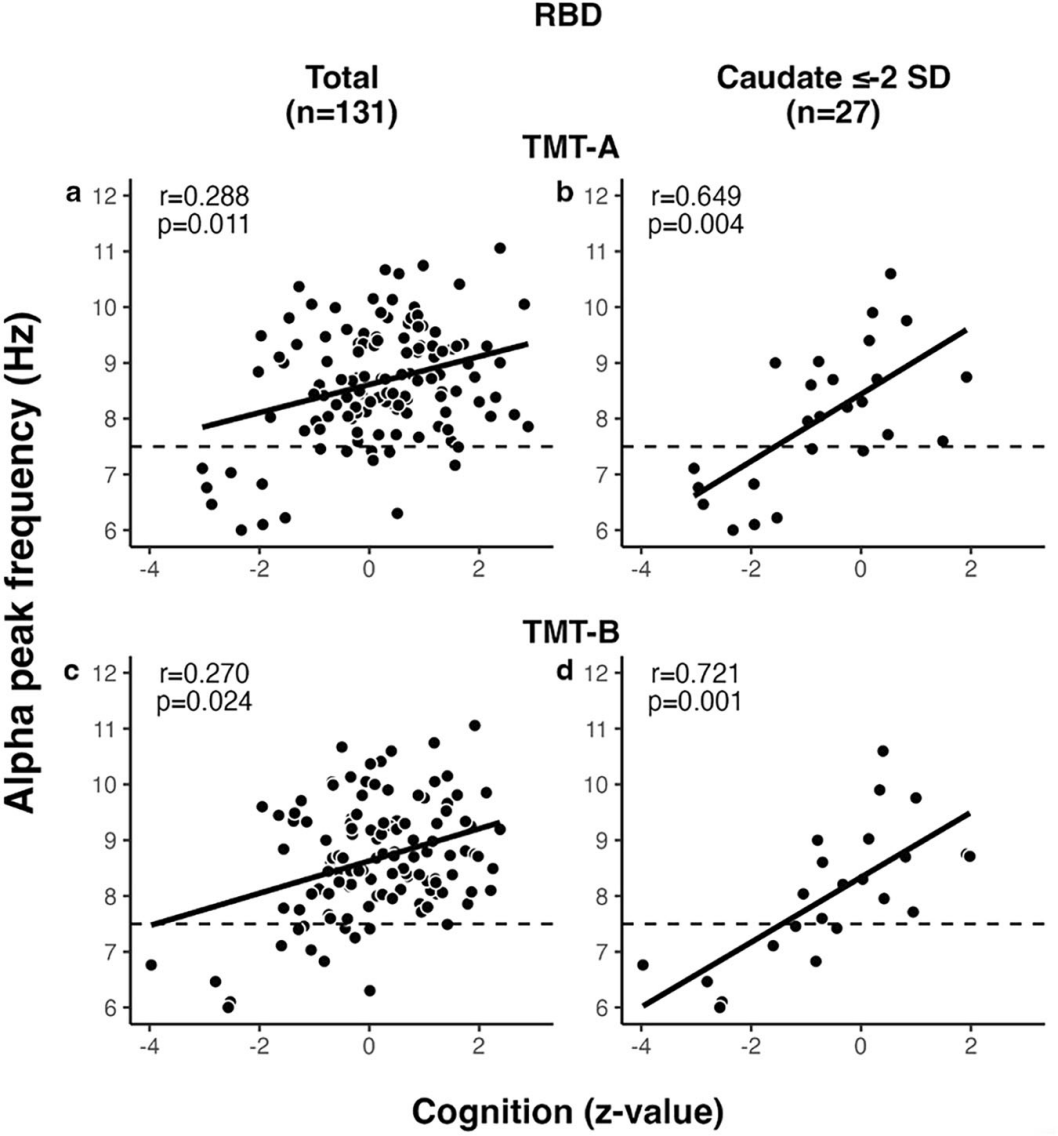
APF vs. cognition. APF correlated significantly with TMT-A and TMT-B in **a/c** RBD patients (*n* = 131), **b/d** patients with most affected caudate ≤−2 (*n* = 27).

In the analysis of APF correlations with CERAD-Plus scores for patients with RBD (*n* = 131; iRBD and RBD-converted patients), significant correlations were observed only for TMT-A and TMT-B (*z*-values; TMT-A: *r* = 0.288, *p* = 0.011; TMT-B: *r* = 0.270, *p* = 0.024; Fig. 4a, c). In patients with RBD who had a DaT-SPECT *z*-value of ≤−2 in the caudate (*n* = 27), the significant correlation between APF and TMT-A and TMT-B were stronger than those observed in the total group of patients with RBD (TMT-A: *r* = 0.649, *p* = 0.004; TMT-B: *r* = 0.721, *p* = 0.001; Fig. 4b, d).

### Impact of APF threshold on correlation with DaT-binding

**Fig. 5:**
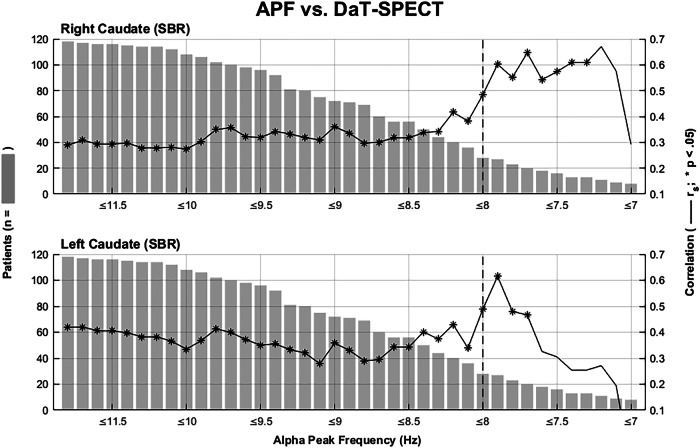
Gradual correlation of APF vs DaT-binding. Starting on the left with all RBD patients included, this figure shows the Spearman´s rank coefficients of the correlation between APF and DaT-SPECT right caudate, left caudate, in subgroups of RBD patients by excluding patients based on their APF with increments of 0.1 Hz. Gray bars show the number of RBD patients included in the correlation. *significant correlation (*p* < 0.05).

Since performing a wake-EEG to determine the APF is a much simpler method and more widely available compared to performing a DaT-SPECT we took a closer look at the correlation between the two and investigated whether there existed an APF threshold beyond which the correlation increased in strength. In Fig. 5 the APF is correlated with the right) and left) caudate DaT-SPECT region showing the correlation coefficients in subgroups of RBD patients with incremental lower APF. What stands out is that the correlation coefficient remains stable in those RBD patients with a “healthy” APF above 8 Hz. For example, excluding patients with an APF faster than 9 Hz does not change the r-value of the correlation much. But as soon as the incremental exclusion of patients passes the 8 Hz threshold the correlation coefficient goes up. This could be observed for correlations with the left and right caudate, but not for the putamen (left/right anterior and posterior putamen). This suggests that an APF below 8 Hz may be indicative of a reduced DaT-SPECT, especially a reduction of DaT-binding in the caudate regions.

## Discussion

To our knowledge, no previous study has focused specifically on the APF when investigating the associations between EEG, cognition and DaT-SPECT in RBD patients. Our analyses show significant correlations between APF and DaT-binding ratios in all studied brain regions in a large single-center cohort of patients with RBD. These correlations were stronger in patients with DaT-SPECT binding ratios indicative of a more advanced pathological state (caudate *z*-values ≤−1.5)^21^ and became stronger in patients with an APF below 8 Hz. The strongest correlations were observed for the caudate region, which is consistent with two established facts: 1. APF slowing is a marker of cognitive decline^10,16^ and 2. impaired dopaminergic function in the caudate is related to cognitive deficits in PD^17–19,22^.

The mean APF in our cohort of patients with iRBD (8.78 Hz) was lower than that of our Non-Syn patients (9.09 Hz), corroborating findings from previous studies with smaller RBD cohorts^11,23^. Additionally, the APF of patients in our cohort who had already converted to overt α-synucleinopathies (PD 7.57 Hz; DLB 6.88 Hz) is in line with previous studies^10,24^ and was lower compared to the prodromal α-synucleinopathy group – patients with iRBD. Thus, when considering our results alongside existing literature, it can be inferred that APF shows a progressive decline from a healthy state through a prodromal state (iRBD) and finally to overt α-synucleinopathy – even more pronounced in patients with DLB.

The APF is known to decrease with healthy aging^16^ and to be a marker of dementia^24^. While APF typically measures around 10 Hz in healthy young adults, it slows to about 9 Hz in healthy older adults (≈70 years old)^25^. This reduction coincides with age-related cognitive decline^16^. To account for these age-related changes, the scores from the CERAD-Plus test battery are corrected for age and expressed as z-values^26^, as are the DaT-SPECT values^27^. Similarly, the CERAD-Plus and DaT-SPECT values are also corrected for sex. And while we found no significant effects of sex, sex differences have previously been reported in EEG changes in patients with PD, with higher functional connectivity impairment found in men, especially in the alpha frequency range^28^. Of particular interest is that this study also showed that these changes in the EEG alpha network correlated with cognition. And this correlation was stronger in early-stage PD patients who had RBD compared to early-stage PD patients without RBD^29^. The underlying mechanism behind this association of EEG alpha changes and cognitive impairment may be cholinergic dysfunction, with studies linking decreased functional connectivity in the alpha frequency with impairments of cholinergic systems in patients with mild cognitive impairment^30^.

Although it is not possible to predict the specific α-synucleinopathy to which patients with iRBD will convert, previous studies have attempted to use EEG slowing to differentiate between PD, DLB and Alzheimer’s disease^10^. Cognitive domains can also provide clues regarding the underlying pathology, with executive functioning being the first cognitive domain to show impairments in PD, followed closely by memory impairments^31^.

Cognitive deterioration has been found to start early in the course of the disease in patients with iRBD, first manifesting as a decline in attention and executive functioning^32^. Similarly, the DaT-SPECT region most affected in PD is the posterior putamen^33,34^, whereas the caudate is more severely impaired in dementia, particularly in DLB^34^. A recent multicenter study showed that while clinical data alone has low predictive value for phenoconversion, combining this data with DaT imaging can predict phenoconversion reasonably well^20^. In our study, we found that the APF correlates more strongly with DaT-SPECT values in the caudate region. This correlation was even stronger in iRBD patients who were in a more advanced prodromal state of the disease, as indicated by a DaT-SPECT *z*-value of ≤−1.5 in the caudate. We also found that there may be an inflection point for the APF at 8 Hz and that APFs below this threshold correlated stronger with specifically the DaT-binding in the caudate region. These findings suggest that APF may become a useful additional marker in iRBD, corresponding to impairments in both cognition and DaT-SPECT.

One previous study examined whether EEG slowing was correlated with DaT-SPECT but found no significant correlations, albeit likely due to its small sample size (*n* = 18)^13^. Additionally, while earlier studies investigating EEG slowing in iRBD in association with cognition have reported APF values, they did not use APF for correlation analysis but instead used EEG power metrics^11–13^. These studies found significant correlations between the EEG slow-to-fast power ratio and executive functioning and demonstrated that EEG metrics could predict the phenoconversion of iRBD over a three-year period^35^.

A limitation of our study is the lack of analysis of EEG power metrics. However, determining EEG power to calculate the slow-to-fast power ratio is more complex and time-consuming than determining the APF, making it less suitable for routine analysis. Another limitation may be the relatively small number of Non-Syn and converted patients in our study as well as the lack of a healthy control group without RBD or other sleep-related or neuropsychiatric conditions, which may have influenced cognitive performance in the Non-Syn group. Of course, to perform radioactive DaT-SPECT in healthy subjects touches ethical implications. Despite these limitations, our findings are consistent with the previous literature^11,13,23,36^. Ultimately, the predictive value of APF for phenoconversion can only be definitively assessed through prospective studies.

In conclusion, APF was significantly correlated with both cognition and DaT-SPECT results in our large single-center cohort of RBD patients. These correlations were strongest for executive functioning – the cognitive domain that is typically the first to be affected in α-synucleinopathies –and for DaT-SPECT caudate regions – the region that is first to be affected in patients with DLB – particularly in patients with iRBD in an advanced prodromal state. Given that PSG recordings are essential for diagnosing iRBD and that APF is relatively simple to determine compared to other EEG slowing metrics, APF can be considered an attractive candidate for use as an additional biomarker in prodromal α-synucleinopathies, both in research and clinical settings.

## Methods

### Patients

In this retrospective cross-sectional observational study, a total of 320 consecutive patients clinically suspected of having RBD who visited the clinic between January 2016 and December 2024 underwent three-night video polysomnography (vPSG). Of these, 245 patients additionally underwent DaT-SPECT imaging and 288 completed neurocognitive testing. After excluding patients with secondary RBD (*n* = 14), those on psychotropic medication (*n* = 87), and for technical reasons (*n* = 30), we categorized the remaining 189 patients into three groups: (1) a control group consisting of patients whose motor behavior during sleep was not attributable to RBD (Non-Syn); (2) patients diagnosed with iRBD; and (3) patients with RBD who had already converted to overt α-synucleinopathies prior to visiting our clinic (see flowchart of Fig. 1). All patients provided written informed consent to participate in the examinations and for the analysis and publication of their anonymized data.

The diagnosis of patients with overt *α*-synucleinopathies (i.e., PD and DLB) was performed by referring psychiatrist/neurologist prior to patients visiting our clinic. RBD, as well as secondary RBD, was diagnosed in our clinic based on polysomnographic diagnostic criteria according to standard American Academy of Sleep Medicine (AASM-ICSD-3) criteria^37^. Secondary RBD was defined as REM sleep without atonia and complex behavior during REM sleep occurring only after respiratory events, or when RBD symptoms clearly had started after antidepressant use (ICSD-3)^37^. Non-Syn patients had reported clinical signs of RBD, but motor behavior during sleep was diagnosed as due to sleep apnea (*n* = 17), periodic leg movements (*n* = 3) and no PSG confirmed sleep disorder (*n* = 14).

### Ethics declaration

All patients gave a written informed consent and the study was approved by the local ethical committee of the Charité University Medicine Berlin, Germany (EA1/138/21) and conformed to the tenets of the Declaration of Helsinki.

### DaT-SPECT

DaT-SPECT was performed to assess presynaptic dopamine transporter binding. SPECT images were acquired approximately four hours after injection of 130–185 MBq [^123^I] ioflupane (FP-CIT, Datscan, GE Healthcare) using a GE Healthcare NM/CT 670 camera. The SPECT data were reconstructed iteratively using HERMES HybridRecon^38,39^, aligned to a template, and automatically analyzed using the latest version of BRASS^TM^ ENC-DAT (EARL) with standard settings for all patients. The specific binding ratio (SBR) was calculated for six different striatal regions (right and left caudate nucleus, anterior putamen, and posterior putamen) using ioflupane uptake in the occipital lobes as the reference for background activity. These results were then corrected for camera- and age-dependencies and compared (*z*-value) with a normal cohort using data from 103 SPECT examinations from the European multicenter database of healthy controls for FP-CIT-SPECT (ENC-DAT)^27^. For a detailed description of the DaT-SPECT procedure, see Kunz et al., 2023^40^.

### Polysomnography

Video-PSGs (vPSG) were conducted over three consecutive nights, capturing EEG, electrocardiogram, electrooculogram, electromyogram (chin and limbs), airflow, respiratory effort, snoring, oxygen saturation, core body temperature, and both video (infrared, 25 frames per second) and audio (44.1 kHz, 16 bit, mono) recordings. Data from the second night was used for analysis to exclude potential adaptation effects from the first night and any effects related to the administration of melatonin, which was initiated on the third night. As a neuropsychiatric sleep laboratory, we prioritize capturing representative sleep patterns in our patients. Therefore, our standard protocol encourages patients to sleep and wake at their preferred times. For this reason, after the PSG setup, the “lights out” and “lights on” times were individually determined based on each patient’s preference. Sleep stages, associated events and polysomnopgraphic diagnostic criteria of RBD were scored in 30 s epochs of the PSG according to standard American Academy of Sleep Medicine (AASM-ICSD-3)^37^ criteria. Because muscle atonia is disturbed in individuals with RBD, we modified the scoring of REM sleep according to the methods proposed by the Montreal group^41,42^. Further details of the standard PSG protocol in our laboratory are described in Kunz et al.^40^.

### Alpha peak frequency

The analysis of the EEG alpha peak was performed using a custom MATLAB script (The Mathworks, Inc., Natick, Massachusetts, USA) on PSG epochs scored as “awake”. Power spectra (bandwidth: 0.1–100 Hz, bin width: 0.1 Hz) were calculated for every successive five-second block without overlap for the O1-A2 and O2-A1 derivations using a fast Fourier transformation (FFT), including the bio-calibration signals prior to lights off. To increase the accuracy of the frequency calculation, each block was extended by five seconds with zero values.

Blocks containing calibration signals, movement artifacts or missing data were excluded from the automatic alpha peak calculation. The classical EEG alpha band (8–13 Hz) was extended downward to 6 Hz because previous research has shown that the dominant peak frequency may slow to the higher theta range in patients who have parkinsonism with dementia (6–13 Hz; hereinafter referred to as alpha)^10,24^.

Blocks containing a clear EEG alpha peak were defined by the criterion that the power at the peak frequency in the alpha range was at least five times higher than the mean power in the 6 to 30 Hz range. To help filter out blocks with sleep stage 1 blocks in which the mean power in the 1 to 6 Hz frequency range was more than twice as high as the mean power in the 6 to 30 Hz range were excluded from further analysis. For each patient, the mean value of all frequency bins across all clear alpha blocks was calculated. The frequency in the 6 to 13 Hz range with the maximum power was then identified as the APF if at least five blocks of clear alpha power were detected.

A visual inspection was performed to verify the presence of alpha activity in the blocks detected by the script, and any artifacts were manually discarded as needed. The visual inspection was performed in all patients, for the entire PSG and always by the same person (SSc). Additionally, the effectiveness of the script was assessed by manually calculating the APF for each awake epoch throughout the night in three patients (with nights containing at least 15 min of wakefulness). The APF values derived from the manual assessment did not deviate by more than 0.15 Hz from those obtained using the script.

### Neurocognitive testing

The Consortium to Establish a Registry for Alzheimer´s Disease - Plus (CERAD-Plus) examination was conducted for 131 of the included RBD patients within four months (0.36 ± 0.84 months; mean ± standard deviation; SD) before or after the vPSG examination. The CERAD-Plus battery consists of 11 tests: Verbal Fluency, Boston Naming Test (BNT), Mini-Mental State Examination (MMSE), Wordlist Learning, Wordlist Recall, Wordlist Recognition, Constructional Praxis, Constructional Praxis Recall, Trail Making Test part A (TMT-A), Trail Making Test part B (TMT-B), and Phonetic Fluency, all of which were administered in their standardized form^43^. Due to visual impairments or language difficulties, TMT-B could not be completed by some patients (*n* = 6). For patients who exceeded the maximum allotted time of five minutes in this test (*n* = 4), a penalty was calculated according to Schmid et al.^43^. The results of the subtests were standardized to account for the influence of age, education level and gender, with scores expressed as *z*-scores^43^.

### Statistical analyses

All statistical analyses were performed using R version 4.3.2 with RStudio 2022.12.0 + 353 “Elsbeth Geranium” Release for Windows. The Shapiro-Wilk test was used to assess the normality of data distributions. Pearson correlations were used to test relationships within groups for normally distributed data, whereas Spearman rank correlations were used for non-normally distributed data. Age correction was applied to the entire cohort using partial correlation. All correlation tests were two-tailed. For comparisons of means, the equality of variances was first assessed using an *F*-test, followed by a *t* test. The p-values were adjusted for multiple comparisons using the Bonferroni-Holm correction. A significance level of *α* = 0.05 was set. Age and sex were included as covariates in all statistical models to control for potential confounding effects, and were removed when they did not change significant results.

## Supplementary information

STROBE Statement—Checklist

## Data Availability

Data are available from the corresponding author upon reasonable request.
